# Elucidating the link between thyroid cancer and mercury exposure: a review and meta-analysis

**DOI:** 10.1007/s11356-024-32031-8

**Published:** 2024-01-26

**Authors:** Alyssa M. Webster, Dylan Pinion, Eric Pineda, Hadeel Aboueisha, Mohammad H. Hussein, Manal S. Fawzy, Eman A. Toraih, Emad Kandil

**Affiliations:** 1 School of Medicine, Tulane University, New Orleans, LA 70112 USA; 2 Medical Education Department, Faculty of Medicine, Suez Canal University, Ismailia, 41522 Egypt; 3 Division of Endocrine and Oncologic Surgery, Department of Surgery, School of Medicine, Tulane University, New Orleans, LA 70112 USA; 4 Department of Biochemistry, Faculty of Medicine, Northern Border University, Arar, Saudi Arabia; 5 Unit of Medical Research and Postgraduate Studies, Faculty of Medicine, Northern Border University, Arar, Saudi Arabia; 6 Genetics Unit, Department of Histology and Cell Biology, Faculty of Medicine, Suez Canal University, Ismailia, 41522 Egypt

**Keywords:** Thyroid, Cancer, Mercury, Risk, Environment, Diet, Review

## Abstract

Mercury (Hg) is a widely distributed and bioavailable metal of public health concern, with many known human toxicities, but data regarding mercury's influence on thyroid cancer (TC) is scarce. Mercury is known to impact several molecular pathways implicated in carcinogenesis, and its proclivity for bioaccumulation in the thyroid suggests a potential modulatory effect. We conducted a literature/systematic review of studies between 1995–2022 intending to define better and establish relationships between these two entities, congregate the evidence for mercury's potential role in thyroid carcinogenesis, and identify populations of interest for further study. Insufficient evidence precludes definitive conclusions on dietary mercury as a TC risk factor; however, several common mechanisms affected by mercury are crucial for TC development, including biochemical, endocrine, and reactive oxygen species effects. Quantitative analysis revealed associations between TC risk and mercury exposure. In three mercury studies, average urine levels were higher in TC patients, with a mean difference of 1.86 µg/g creatinine (95% CI = 0.32–3.41). In two studies investigating exposure to elevated mercury levels, the exposed group exhibited a higher risk of developing TC, with a relative risk of 1.90 (95% CI = 1.76–2.06). In three thyroid tissue studies, mercury levels (ppm) were higher in TC patients, averaging 0.14 (0.06–0.22) in cancerous cases (N = 178) and 0.08 (0.04–0.11) in normal thyroids (N = 257). Our findings suggest an association between mercury exposure and TC risk, implying a possible predisposing factor. Further research is necessary to reveal the clinical relevance of dietary and environmental mercury exposures in TC pathogenesis.

## Introduction

Despite its high survival rate, thyroid cancer (TC) has garnered considerable interest due to its rapidly escalating incidence in the last two decades, averaging a 3% annual increase between 2000–2013 (Lim et al. 2017). Since 2015, TC incidence has stabilized, attributed mainly to advancements in American Thyroid Association (ATA) management guidelines and improved early-stage tumor detection and targeting (Pereira et al. 2020). Despite these encouraging statistics, TC mortality and diagnoses of the advanced disease continue to rise, suggesting an authentic trend of increased incidence (Megwalu and Moon 2022). TC remains the most prevalent endocrine malignancy, and its disease burden has escalated (Deng et al. 2020).

Mercury (Hg), a heavy metal, poses significant public health concerns due to its toxicity and widespread human exposure. Mercury is found in seafood, household items, medical equipment, and cosmetics, among other sources, and it is a common occupational exposure hazard (Bjørklund et al. 2017). Pollution and volcanic eruptions contribute to its environmental presence in soil, water, and atmosphere (Rice et al. 2014; Gworek et al. 2020). Mercury is known to be neurotoxic at high doses and toxic to cardiovascular, immune, and endocrine systems (Rice et al. 2014), and its potential role in carcinogenesis at low doses continues to draw attention.

A review by Skalny et al. (2022) examined mercury's carcinogenic effects and identified numerous potential mechanisms through which it may promote carcinogenesis, particularly at low doses. Increased body mercury levels, especially in the hair, nails, blood, and urine, were associated with a higher risk of non-melanoma skin cancer, basal cell carcinoma, melanoma, squamous cell carcinoma, prostate cancer, renal cell carcinoma, and elevated lung cancer mortality. Increased dietary mercury intake correlated with colorectal cancer risk, but the review found no association between body mercury burden and risk for glioma, breast cancer, or TC. An analysis of National Health and Nutrition Examination Survey (NHANES) data from 1999–2014 showed no association between blood mercury levels and cancer mortality (Skalny et al. 2022).

These findings partially contradict information from the US Environmental Protection Agency (EPA), which states that while there is not enough data to make a definitive conclusion, no human studies have linked mercury exposure to cancer. High doses of certain forms of mercury have caused increased tumor rates in rats and mice (EPA, 2023). However, per the EPA Cancer Guidelines published in 2005, "mercury and methylmercury are not likely to cause cancer in humans" (US Environmental Protection Agency 2005). Though data remains limited, accumulating evidence in recent years suggests a more active role for mercury in the pathophysiology of an array of malignancies (Skalny et al. 2022).

Concerning TC specifically, Skalny et al.'s review included only three relevant studies, all of which failed to demonstrate an association between mercury and TC development (Skalny et al. 2022); however, the opposite is true for several studies not included in the paper which have found an association between mercury exposure and TC risk (Kim et al. 2022; Zaichick 2022; Correia et al. 2020; Zhang et al. 2019; Malandrino et al. 2016). Furthermore, mercury has a proclivity to accumulate in thyroid tissue and affect thyroid hormone synthesis, which has known associations with TC development (Marotta et al. 2020). To our knowledge, no existing literature exists that consolidates these findings comprehensively. Owing to these conflicting findings and the paucity of complete data, the current review thoroughly examines the evidence for mercury as a potentially significant factor in TC development and carcinogenesis.

## Materials and methods

We included a total of 53 unique studies in our final review. We first conducted a literature review based on a keyword search in PubMed and Google Scholar, targeting titles, abstracts, and full texts containing variations of mercury, including "mercury, "Hg," "methylmercury," and "MeHg," and thyroid cancer including "thyroid cancer," "thyroid carcinoma," "thyroid tumor," and "thyroid nodule.", which returned over 1,200 articles. After a preliminary review of the literature and title screening, articles of interest were flagged, and three independent investigators performed separate literature reviews searching for an association between environmental and occupational mercury exposures and TC, including the terms "environment," "emissions," "atmosphere," "inhalation," and "occupation," as well as the association between dietary mercury and TC including the terms, "dietary," "ingest," "fish consumption." We searched for an association between mercury in cosmetics and TC using the terms "cosmetic," "makeup," and "product." Additionally, we reviewed the available literature on the impact of mercury on biochemical and molecular pathways involved in thyroid carcinogenesis, including the terms "carcinogenesis," "cancer development," "cell proliferation," and "pathway." After finalizing the literature review, we identified 45 unique studies examining the role of environmental and dietary mercury in TC development and the carcinogenic mechanisms potentially influenced by mercury, which are also considered crucial for TC development. Of these, three were discussed again in our quantitative analysis (Kim et al. 2022; Malandrino et al. 2016; Zidane et al. 2019). Revisions and supplementary searches were conducted as needed by a fourth investigator to ensure completeness.

An Ingenuity Pathway Analysis was performed to identify novel biomarkers and significantly enriched pathways and networks associated with mercury-induced TC. The purpose of this analysis was to predict potential upstream regulators and downstream effects and to identify possible therapeutic targets. The IPA software combines a comprehensive, curated database of biological interactions and functional annotations derived from intricate molecular datasets, including transcriptomics, proteomics, and metabolomics data in published scientific literature.

**Fig. 1:**
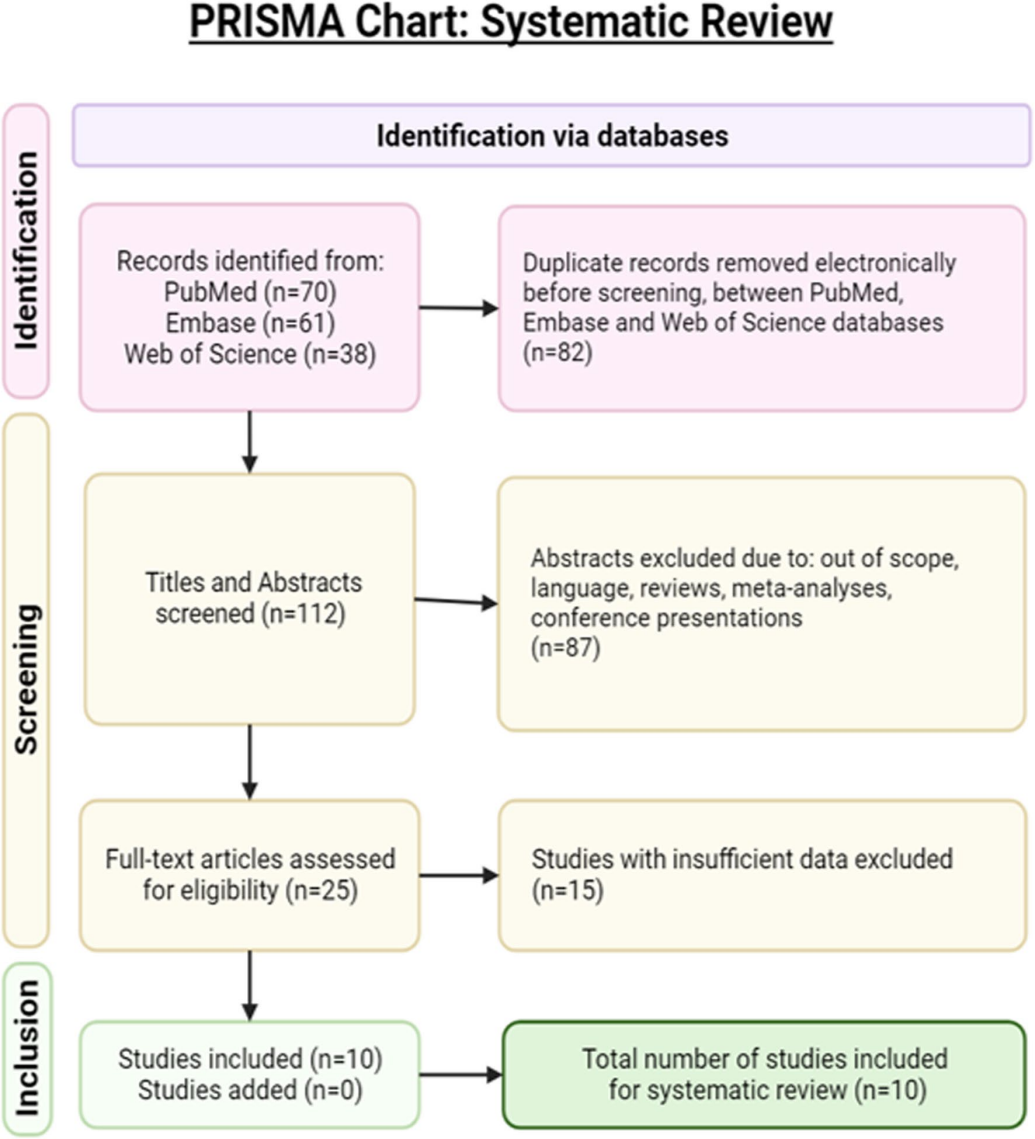
PRISMA diagram

For the quantitative data, two independent investigators performed a systematic review following PRISMA guidelines in PubMed, Embase, and Web of Science, with the search terms "thyroid" in the title AND "(cancer OR carcinoma OR tumor OR nodule)" anywhere in the text AND "(mercury OR Hg OR methylmercury OR meHg)" anywhere in the text. This search resulted in 112 unique studies, which were screened for relevance, including studies that evaluated tissue mercury levels and rates of thyroid cancer while excluding studies with non-human subjects, studies lacking data on tissue mercury levels, studies focused on non-thyroidal cancers, and studies focused on non-cancerous thyroid conditions. This left 25 studies for full-text review, of which 15 were excluded for improper study design or insufficient data, and ten were included in the final review, as shown in the PRISMA diagram in Fig. 1. These included cohort studies and case series, which contained data on urinary or tissue mercury levels and TC incidence, spanning 1995–2022. We analyzed the quantitative data on urinary or tissue mercury in the context of TC risk and evaluated the potential association between mercury exposure and TC. The untransformed (raw) means for urinary mercury were compared between TC and non-TC cohorts, and for studies with tissue measurements, the mean mercury levels were compared in cancerous versus non-cancerous thyroids using the inverse variance method and the DerSimonian-Laird estimator. In the studies with tissue measurements, the mean mercury levels were compared in cancerous versus non-cancerous thyroids. The inverse variance method and DerSimonian-Laird estimator were utilized.

Finally, we compared global mercury emissions to global TC incidence using the Arctic Monitoring and Assessment Program (AMAP)/United Nations Environment Program (UNEP) 2013 Global Mercury Modelling Report and 2020 GLOBOCAN database.

## Review/meta-analysis results and discussions

### Quantitative analysis

A comprehensive search of the literature yielded nine relevant studies containing quantitative data linking mercury exposure to the risk of TC: five urine studies (Kim et al. 2022; Liu et al. 2021; Correia et al. 2020; Zhang et al. 2019; Malandrino et al. 2016), three tissue studies (Zaichick 2022; Chung et al. 2016; Zaichick et al. 1995), one blood level study (Chung et al. 2016), and one study using fingernail measurements (Zaichick 2022). In an epidemiological study of nearly 3 million individuals, there was approximately a 90% increased risk of TC in the exposed group (Malandrino et al. 2016). Of the nine studies, seven found some level of association between increased mercury exposure and TC risk (Kim et al. 2022; Zaichick 2022; Liu et al. 2021; Correia et al. 2020; Zhang et al. 2019; Zidane et al. 2019; Malandrino et al. 2016), while two did not (Zidane et al. 2019; Chung et al. 2016). Among the five urine studies, three (Kim et al. 2022; Liu et al. 2021; Zhang et al. 2019) compared mean urine mercury levels in TC cases and non-TC controls, and two examined the risk of TC in mercury-exposed versus non-exposed groups (Correia et al. 2020; Malandrino et al. 2016).

#### Urine mercury comparisons

**Fig. 2:**
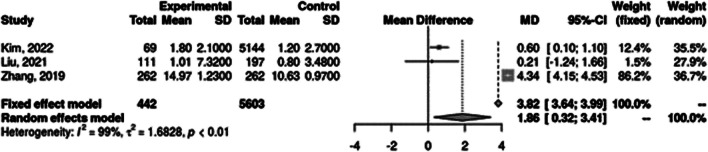
Mean urine mercury in TC + and TC- groups. Mean difference (MD) was calculated using the inverse variance method and Hunter-Schmidt estimator for tau^2

**Fig. 3:**
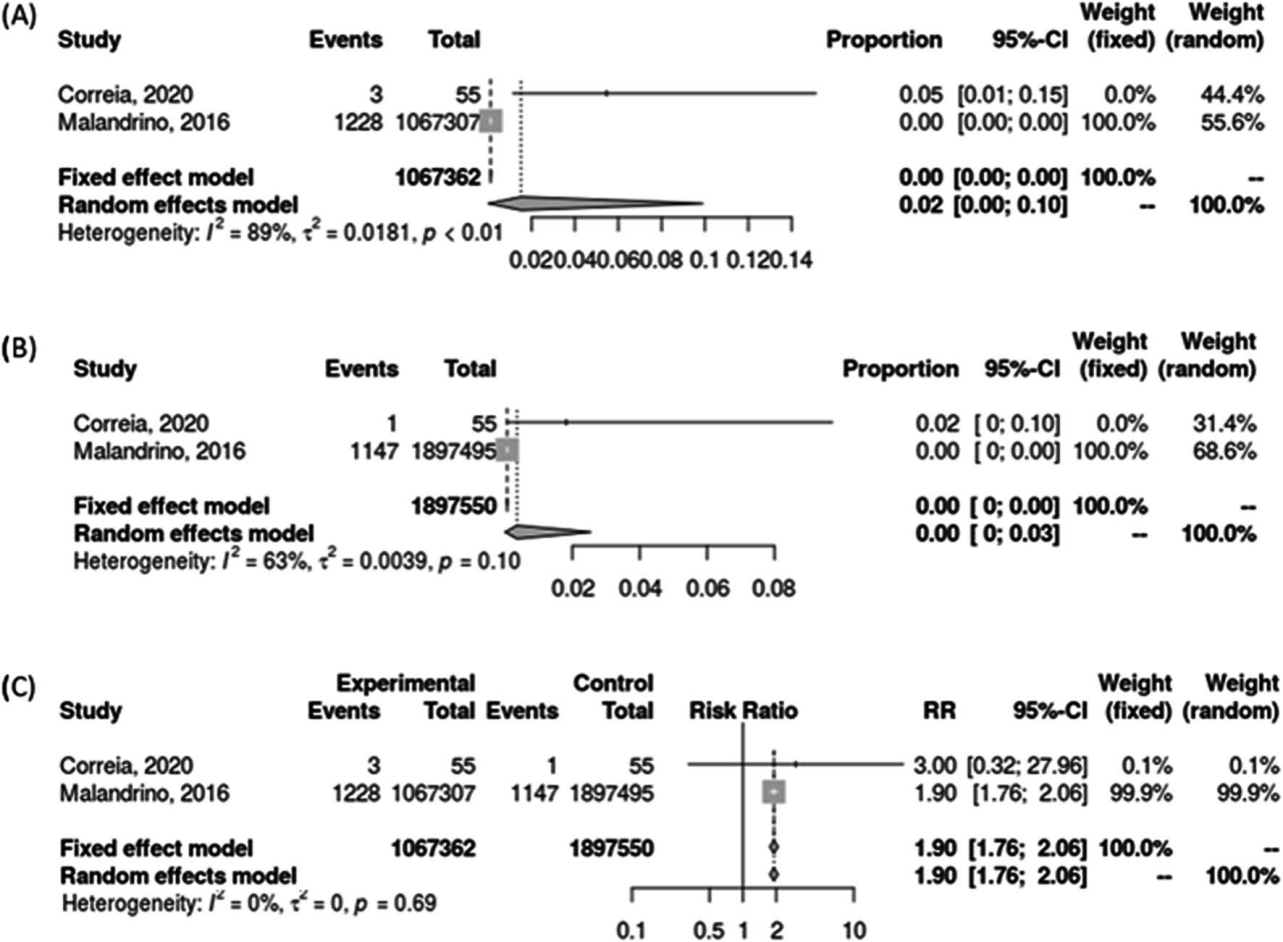
Risk of TC in exposed groups with very high urine mercury levels in a volcanic environment or occupational setting. (A) The proportion of TC risk in the exposed group. (B) The proportion of TC risk in the non-exposed group. (C) The relative risk of TC in exposed vs. unexposed groups

In three studies, average urine mercury was found to be higher in patients with TC by a mean difference of 1.86 ug/g creatinine (95% CI = 0.32 – 3.41) (Fig. 2). In one volcanic study and one study involving occupational exposure to high mercury levels, the risk of developing TC in the exposed group was higher, RR = 1.90 (95% CI = 1.76 – 2.06) (Fig. 3).

#### Thyroid tissue comparisons

**Fig. 4:**
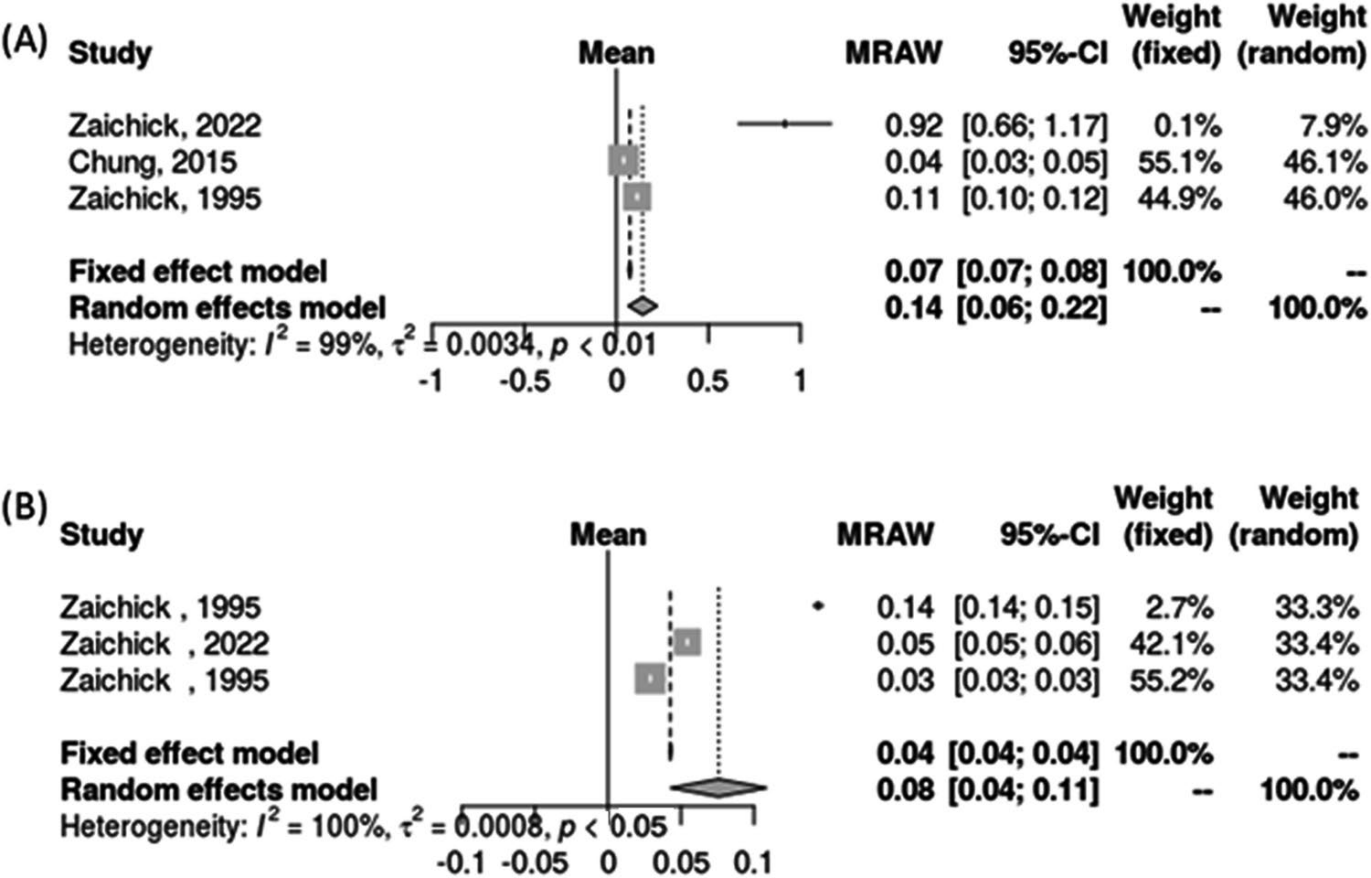
Raw means of tissue mercury levels in (A) cancer and (B) non-cancer thyroid tissues using the inverse variance method and DerSimonian-Laird estimator

In three studies involving thyroid tissue, mercury levels (ppm) were higher in patients with TC, with an average of 0.14 (0.06 – 0.22) in TC (N = 178) and 0.08 (0.04 – 0.11) in normal thyroids (N = 257) (Fig. 4).

### Locoregional exposure to mercury emissions and TC incidence rates

We further investigated the potential of environmental mercury exposure as a risk factor for TC by comparing mercury exposure through anthropogenic emissions from the United Nations Environment Program (UNEP) 2013 Global Mercury Modelling Report to global TC incidence from the 2020 GLOBOCAN database (Table 2).

Some regions were identified with high mercury emissions and TC incidence, and vice versa. For North America, the emissions from stationary combustion sources, industrial sources, and intentional use and product waste are 32.4, 34.8, and 41.1 tons/year, respectively, among the highest emissions identified in the UNEP report. This region also has the highest age-standardized incidence rate of TC at 12.4 per 100,000 people and a mortality rate of 0.3 per 100,000. East Asia also follows this trend as it has the highest mercury emissions, with 56.9, 71.6, and 42 tons/year from the three sources. It has the second-highest TC incidence at 11.5 per 100,000 people and a mortality rate of 0.4 per 100,000. South America is also among the regions with high mercury emissions and TC incidence. In contrast, Southeast Asia has lower pollution levels (7.9, 14.1, and 16.4 tons/year from the same sources, respectively) and correspondingly lower TC incidence and mortality rates (4.4 and 0.63 per 100,000 people, respectively). Additionally, Central America and Europe were among the regions with low levels of both mercury emissions and TC incidence.

Other regions were outliers for this trend. South Asia has relative emission levels of 27.7, 21.2, and 17.9 tons/year, respectively, and the lowest TC incidence of 1.6 per 100,000 people. Similarly, Africa had the highest emissions at 33.7, 50, and 80.7 tons/year, respectively, and the second lowest TC incidence at 2 per 100,000 people. These outliers may be secondary to genetic, cultural, diagnostic, and environmental confounding factors causing an actual or artificial reduction in TC incidence.

**Fig. 5:**
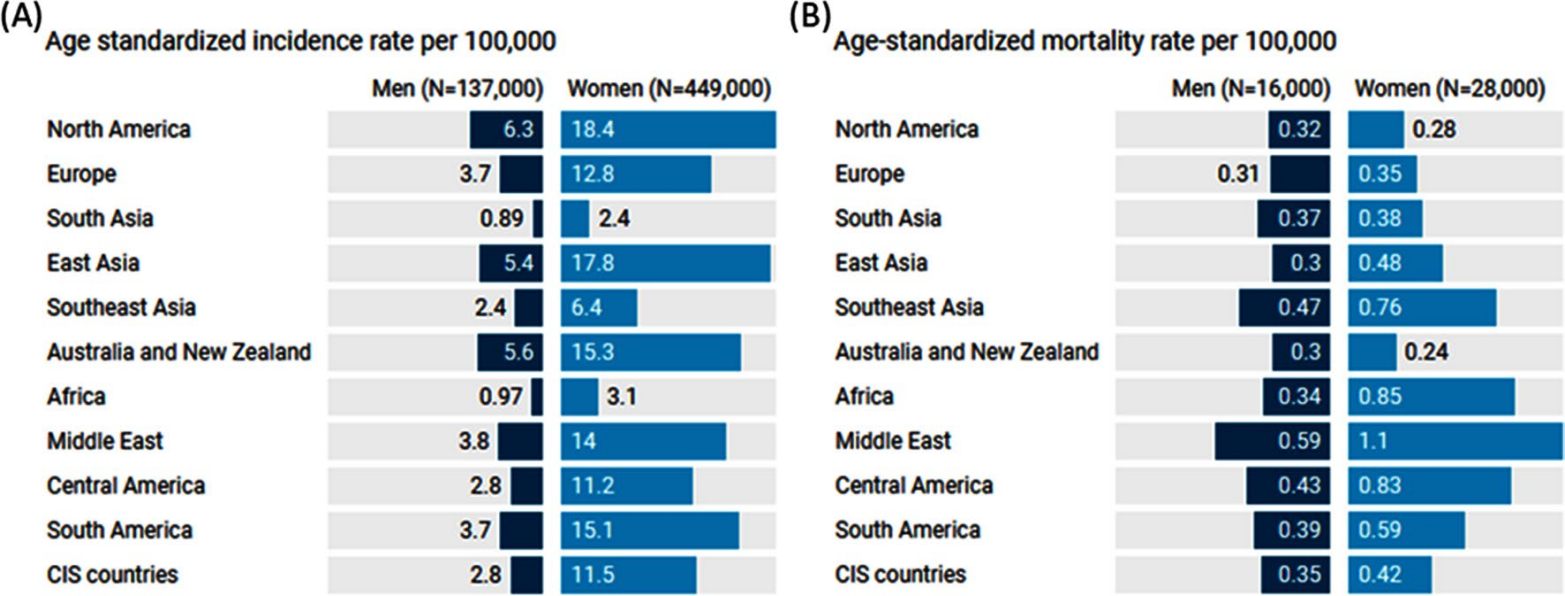
Gender-specific rates in thyroid cancer across different regions from the 2020 GLOBOCAN database. (A) Age-adjusted standardized incidence rate (SIR) per 100,000 in men and women. (B) Age-adjusted standardized mortality rate (SMR) per 100,000 in men and women

Figure 5 provides specific incidence and mortality rates for men and women and highlights the striking differences in TC incidence. For instance, in North America, there is a roughly three-fold increased incidence for women at 6.3 and 18.4 per 100,000 men and women, respectively, while the mortality rates are 0.32 and 0.28 per 100,000 men and women. In South America, the difference is nearly five-fold, with incidence rates of 3.7 and 15.1 per 100,000 men and women, respectively. These gender-specific rates could suggest that gender-based biological differences or social factors could play a role in TC incidence and mortality, and further investigation is needed to explore these disparities.

Of note, the Federated States of Micronesia, Melanesia, and French Polynesia were not represented in the UNEP report but had strikingly high TC incidence and mortality for women, with the incidence in French Polynesia being the highest of any region at 23.3 per 100,000 women, and mortality rates the highest in Melanesian women at 2.8 per 100,000. This is especially striking compared to the regions with the lowest incidence and mortality, such as Africa and South Asia (Pizzato et al. 2022). Although this region was not included in the UNEP report, it is notable for the high incidence rates of many cancers due to sociocultural factors and environmental radiation exposure (Pineda et al. 2023). However, multiple studies have also shown evidence of significant mercury exposure in this population, mainly through diet (Hightower et al. 2006; Zidane et al. 2019).

## Literature review

### Natural and anthropogenic mercury emissions: global overview and relative contributions

Global natural mercury emissions are estimated to be roughly 5,207 tons per year, with the largest share (roughly 36%) coming from the oceans (Sundseth et al. 2017). The exact contribution of volcanoes and geothermal activity is unknown and difficult to assess; however, mercury from these sources may account for a sizable portion of all primary atmospheric mercury emissions (Edwards et al. 2021). Volcanic emissions account for an estimated 20–40% of natural mercury emissions, mostly from small, sporadic eruptions (Pyle and Mather 2003). Many areas of high volcanic and geothermal activity have been associated with elevated levels of mercury in the soil and air, including Hawaii, Iceland, the western United States, and New Zealand (Gworek et al. 2020).

In contrast, anthropogenic sources of mercury are estimated to emit roughly 1,960 tons per year, the majority of which is attributed to artisanal and small-scale gold mining (ASGM) and the combustion of coal and other fossil fuels (Sundseth et al. 2017). As a result, individuals living in low-income and developing nations are at higher risk of mercury exposure and its downstream health consequences (Ruggieri et al. 2017). Both natural and anthropogenic mercury emissions contribute to human exposure to inhaled and ingested mercury in different amounts based on location and environment.

### Inhalation exposure to mercury: atmosphere, occupational settings and dental amalgam

Inhalation, the most direct and bioavailable route of exposure to mercury due to its high lipophilicity, is known to occur via atmospheric, occupational, and dental amalgam exposure (Taux et al. 2022). Although overall atmospheric mercury levels are typically low (Fawell et al. 2005), exposure to higher doses may occur in occupational settings and locoregionally due to anthropogenic mercury pollution and volcanic activity (Budnik and Casteleyn 2019; Taux et al. 2022; Pyle and Mather 2003). Additionally, amalgam dental fillings emit mercury vapor at 2–28 ugs per facet surface per day, of which 80% is absorbed (Bernhoft 2012). Dental amalgams also pose a significant concern for mercury emissions during the cremation process due to the high heat and combustion used, and it has been suggested that this may be a contributing factor in the increased incidence of congenital anomalies seen in individuals living in proximity to crematoriums (Tibau and Grube 2019).

### High mercury concentration in soil leading to inhalational and dietary exposure

Mercury may also be present at high concentrations in the soil of polluted regions from natural and anthropogenic sources, leading to both inhalational and dietary exposure. Emissions from land sources are challenging to measure but are estimated to account for roughly 1600–2500 Mg/year (Gworek et al. 2020). We encountered two studies evaluating the correlation between elevated tissue mercury levels due to environmental factors and TC, which are discussed later in this review in the quantitative analysis. Of these, one South Korean study on residents living near industrial complexes found a significant association between elevated urine mercury and TC (HR = 2.11 [CI 1.11–4.03] in T3 vs. T1, p for trend = 0.023) (Kim et al. 2022). In a volcanic study, the authors assessed lichen mercury levels as a surrogate for aquatic and atmospheric mercury levels. They found a higher TC incidence in the volcanic study population of 18.5/10^5^ residents (95% CI = 16.2–20.8) compared with 9.6/10^5^ residents (8.4–10.8) in the matched control population, which was associated with significantly higher urine and aquatic mercury levels in the volcanic group, as well as higher mercury levels in the lichen although this was not statistically significant (Malandrino et al. 2016). However, the exact contribution of soil, aquatic, and atmospheric emissions in both studies remains unclear.

### Dietary exposure to mercury: the risks of seafood consumption and regulatory limits

The US Food and Drug Administration (FDA) has identified dietary exposure to heavy metals, including mercury, as a significant public health concern. Methylmercury (MeHg) represents the most concerning form of mercury; toxicological studies have illustrated its capacity to cross both the placental and blood–brain barriers. Various US regulatory agencies have established reference values delineating daily maximum safe exposure thresholds, reporting limits from 0.1 µg/kg/day to 0.3 µg/kg/day for MeHg exposure (Wong et al. 2022). Typically, mercury levels in rain and groundwater are too low to affect drinking water, although some areas that rely on well water may have dangerously high mercury levels due to contamination. Thus, in non-occupationally exposed groups, the primary route of mercury exposure in the human diet arises from aquatic environments and seafood consumption (Fawell et al. 2005). In aquatic ecosystems, mercury is easily transformed into toxic methyl mercury (MeHg) and may be present locally at high levels due to natural emissions and anthropogenic mercury release (Mahale et al. 2017). Although mercury concentrations in soil and freshwater are low, biomagnification can result in MeHg concentrations in the food chain that are 1–6 orders of magnitude higher than environmental detection levels (Nogara et al. 2019). Additionally, multiple US studies have attributed higher nail and blood mercury levels to increased seafood consumption in some populations (Buchanan et al. 2015; Rees et al. 2007).

To our knowledge, only two studies have investigated the carcinogenic risk of dietary mercury, with mixed results. The French Polynesian population exhibits the highest global TC mortality and incidence rates, and mercury has been detected in their fisheries, including lagoon fish, sea fish, and giant clams, with mean levels measuring 0.43, 0.38, and 0.18 µg/g, respectively. A study of 229 patients with TC and 373 healthy controls from French Polynesia did not reveal significant associations between dietary mercury and TC risk (OR 0.97, 95% CI: 0.41 – 2.29) (Zidane et al. 2019). In contrast, a South Korean study demonstrated a significant association between elevated mercury intake through seafood consumption and colorectal cancer in both men and women. Among 923 patients with colorectal cancer and 1,846 controls, a 2–threefold increased risk was observed across groups with varying mercury intake (OR: 2.57; 95% CI: 2.03, 3.27 for highest compared with lowest tertile; *p* for trend < 0.0001) (Kim et al. 2020).

Some studies have found evidence for a higher incidence of TC in coastal regions with greater seafood consumption, and a few epidemiological studies have demonstrated associations between shellfish consumption and TC. The National Health Screening Service (NHSS) in Norway analyzed data from 60,000 Norwegians who completed a health questionnaire survey and found a higher risk of TC in regular consumers of cod-liver oil, fish liver, or fish sandwich-spread compared to irregular or nonusers (OR 1.48, 95% CI: 0.86 – 2.52) (Glattre et al. 1993). A study in Hawaii reported analogous findings, indicating higher seafood consumption in both men and women (OR_men_ 2.2, (95% CI: 0.7 – 7.0), OR_women_ 1.2, (95% CI: 0.6 – 2.2)) with TC compared to healthy controls (Kolonel et al. 1990).

Indirect associations of mercury intake through seafood consumption might offer insights into TC risk; however, the current body of evidence remains inconclusive and constrained by study type and design. Most data on dietary impacts of mercury or seafood and TC stem from case–control studies. One meta-analysis reported opposing associations linking seafood consumption with lower rates of TC (OR 1.01, 95% CI: 0.86 – 1.19, I^2^ = 51.4%) after examining 13 case–control and 4 cohort studies (Hong and Myung 2023). The study associations lacked statistical significance, underscoring the limitations of available data.

### Mercury contamination in cosmetic products: risks and FDA guidelines

It has been established that mercury is a known contaminant and additive in cosmetic products, with the FDA reporting a prevalence of mercury in anti-aging and skin-lightening products in December of 2022. The current FDA guidelines from February 2022 allow for a maximum of 65 ppm in eye creams only when there are no other safe or effective alternative preservatives, and all other products should not exceed trace 1 ppm (FDA-cite). A systematic review assessing mercury levels in cosmetic products and downstream health effects found exceptionally high mercury levels in skin-lightening products, which was associated with elevated tissue mercury levels. For users of lightning cream, 65% had blood mercury levels above the reference value of 10 ug/L. Additionally, 70.1% of cream users had hair mercury concentrations above 5 ug/g compared to non-cream users, of whom only 0.7% had hair mercury concentrations above 5 ug/g. Many of the individuals in the study with potential mercury exposure reported symptoms of mercury intoxication, including nervousness, headaches, fatigue, weakness, depression, insomnia, and memory loss; however, the incidence of thyroid or other cancers was not reported (Bastiansz et al. 2022).

The link between the use of mercurated cosmetic products and the prevalence of TC is of interest to the present study, but there is scant evidence available to assess the relationship with TC. Given well-known gender discrepancies in TC prevalence and high tissue mercury levels in users of skin-lightening creams (Bastiansz et al. 2022), it would be prudent to investigate the use of cosmetic products as a potential driving factor in thyroid carcinogenesis in the future.

### Occupational hazard of mercury in the gold mining and dental industries

Mercury exposure is an occupational health concern, particularly in professions with substantial chemical and heavy metal exposure and may lead to a higher risk of TC and other malignancies (Skalny et al. 2022). ASGM constitutes a primary source of mercury emissions, with substantially higher mercury concentrations in body fluids and tissues among gold miners than average, which was well established in a systematic review by Taux et al. (2022). Such concentrations often cause overt symptoms of mercury toxicity, including dementia, tremors, cutaneous manifestations, and sexual dysfunction. Laboratory analyses have found indicators of thyroid dysfunction in this population, including elevated thyroid-stimulating hormone (TSH) and diminished triiodothyronine (T3) and thyroxine (T4) levels (Taux et al. 2022).

Dental practitioners may also experience heightened mercury exposure due to their proximity to dental amalgams, especially those with considerable copper content, which exhibit reduced stability and augmented mercury emissions (Tibau and Grube 2019). To our knowledge, the extant literature has not revealed any discernible health ramifications from mercury exposure among dental workers, nor has it demonstrated increased tissue mercury in these individuals. However, mercury emissions from dental amalgam fillings pose an environmental concern due to the high heat used during the cremation process. Empirical investigations have demonstrated elevated hair mercury concentrations among crematorium workers, while the increased incidence of stillbirth, anencephaly, and other congenital anomalies has been observed among individuals residing in proximity to crematorium facilities (Tibau and Grube 2019).

## Biochemical mechanisms which are known to be important in mercury and TC

### Disruption of cellular signaling pathways

The activation of RAS (Rat sarcoma virus; a small GTPase protein implicated in cellular signal transduction) and its downstream signaling cascade plays a crucial role in both the cellular effects of mercury exposure and the development of various forms of TC. RAS activation triggers two pathways, namely MAPK (mitogen-activated protein kinase) and PI3K (phosphoinositide 3-kinases), which are cell-signaling cascades implicated in thyroid carcinogenesis. Activating mutations of these pathways have been identified in several types of TC, including 43% of papillary thyroid carcinomas (PTC), 10% of follicular thyroid carcinomas (FTC), 5–28% of poorly differentiated thyroid carcinomas (PDTC), 9–27% of anaplastic thyroid carcinomas (ATC), and 13–24% of sporadic medullary thyroid carcinomas (MTC) (Prete et al. 2020).

MAPK activation following mercury exposure has been documented in Human Embryonic Kidney (HEK) and thyroid cell lines (Hao et al. 2009; Maggisano et al. 2020). In HEK293 cells, exposure to low-dose mercury was associated with increased phosphorylation of extracellular signal-regulated kinase (*ERK*) and c-Jun N-terminal kinase (*JNK*). In these cells, ERK activation was critical for the responses following HgCl2 treatment, with ERK inhibitors blocking the mercury-induced cellular proliferative response (Hao et al. 2009). Mercury-exposed thyroid cells also faced growth promotion and proliferation, with an increased cell population in the G2/M phase and elevated expression of phosphorylated *ERK* compared to controls (Maggisano et al. 2020). The concept of MAPK activation and cancer extends to TC, particularly concerning two receptor tyrosine kinases, RET and NTRK. RET/PTC fusions, RET/NTRK fusions, RET activating mutations, RAS point mutations, and BRAF point mutations all serve as markers in PTC, FTC, PDTC, ATC, and MTC, due to their promotion of increased MAPK activity and its downstream signaling (Prete et al. 2020).

PI3K signaling is another pathway implicated in both the carcinogenic mechanisms of mercury and the development of sporadic TC. Specifically, the mercuration of phosphatase and tensin homolog (*PTEN*) in SH-SY5Y cell lines demonstrated subsequent inhibition of *PTEN,* promoting Akt/CREB signaling activation and cell survival (Unoki et al. 2016). Similar mechanisms have been identified in sporadic thyroid carcinoma, inhibiting PTEN mutations in 15% of ATC and 4% of PDTC (Prete et al. 2020). Although activating mutations of the *AKT1* gene in TC are rare, they have been reported in 1% of PTC, 1 – 2.6% of FTC, and 0 – 3% of ATC (Prete et al. 2020).

### Disruption of thyroid hormone endocrine pathway

Meta-analyses have revealed the disruptive effects of mercury on the thyroid hormone-hypothalamic–pituitary–adrenal (HPA) axis, showing that it leads to either increased thyroid-stimulating hormone (TSH) or reduced triiodothyronine (T3) and thyroxine (T4) levels (Steinhaus et al. 2021; Marotta et al. 2020). While T3 and T4 levels have varied across studies, the overall association between mercury and elevated TSH levels or low T3/T4 levels suggests a possible reflexive increase in TSH (Marotta et al. 2020). Previous cohort studies and reviews have established a statistically significant relationship between high serum TSH and TC (OR_TSH 0.6–0.89_ = 2.01 (95% CI: 1.46–2.77; p < 0.0001), (OR_TSH 1.6–3.4_ = 4.29 (95% CI: 3.17–5.08; p < 0.0001)) (Fiore et al. 2009) and suggested that increased TSH levels stemming from environmental exposures can affect the proliferation potential of thyroid cells, promoting an increased susceptibility to malignant transformation (Boelaert 2009).

### Pro-oxidative effects and inhibition of DNA repair mechanisms

Mercury exposure has been found to elevate reactive oxygen species (ROS) levels, in part through NADPH oxidase activation (Mohammadi-Bardbori and Rannug 2014; Buha et al. 2021), which can lead to cell proliferation. The upregulation of ROS is also facilitated by mercury-induced inhibition of antioxidants like catalase and glutathione (Skalny et al. 2022). ROS production may induce Nrf2 signaling, inhibiting cellular apoptotic mechanisms (Buha et al. 2021; Tinkov et al. 2021). PTC typically stains strong or moderate for Nrf2 on immunohistochemistry and has positive Nrf2 RNA, whereas benign thyroid tissue stains negatively and has undetectable Nrf2 RNA, and its transcriptional functions appear to promote thyroid cell survival (Ziros et al. 2013). Mercury exposure and subsequent ROS production have been linked to DNA structural damage, including forming OH adducts, 8-OHdG, double-strand breaks, and comet formation (Skalny et al. 2022). Mercury exposure is also associated with increased evidence of DNA damage in cells exposed to exogenous hydrogen peroxide (Wyatt et al. 2017), indicating a role for mercury in cellular ROS production and subsequent DNA damage.

Mercury exposure may also affect DNA repair mechanisms. High-dose mercury exposure in human astrocytes inhibited PARP-1, DNA ligase III-alpha, and XRCC1 (Pieper et al. 2014). Similarly, DNA repair efficiency, as measured by single-cell gel electrophoresis, was significantly reduced in human lymphocytes following occupational exposure to mercury vapor (Cebulska-Wasilewska et al. 2005).

### Summary of findings and ingenuity pathway analysis

We analyzed the molecular pathways connecting mercury exposure to the development of TC. A thorough understanding of these mechanisms is essential for identifying potential risk factors, developing effective preventive measures, and devising targeted therapies for TC associated with mercury exposure. Table 1 presents various pathways in the literature relevant to mercury exposure and TC, emphasizing the complex interplay between these factors in disease progression.

**Table 1:** The key pathways and their relevance to both mercury exposure and TC

Pathway	Hg relevance	TC relevance	Mechanism
RAS/MAPK/PI3K	Hg – > RAS activation	H-RAS, N-RAS, K-RAS activating mutation in TC	RAS activation– > MAPK and PI3K activation downstream
MAPK/ERK	Hg – > MAPK activation	RET/PTC fusion, RET activating mutation, BRAF mutations in TC	MAPK activation– > ERK phosphorylation
PTEN/Akt/CREB	Hg – > PTEN inhibition	PTEN inhibiting mutations, AKT1 activating mutation in TC	Akt/CREB activation
Endocrine/HPA axis	Hg – > Low T3/T4; Elevated TSH	High TSH– > thyroid cell proliferation in TC	Cell proliferation stimulated by TSH
ROS/Nrf2	Hg – > ROS production– > Nrf2 activation	Nrf2 signaling – > PTC	Nrf2 signaling– > Apoptosis inhibition
ROS/ oxidative stress	Hg – > ROS production– > DNA damage	Oxidative stress– > DNA damage– > carcinogenesis	DNA damage
DNA repair	Hg – > Inhibition of DNA repair proteins (PARP-1, DNA ligase III-alpha, XRCC1)	Inability to repair DNA damage– > carcinogenesis	DNA damage

Hg = mercury; RAS/MAPK/PI3K = cell signaling cascades; ERK = extracellular signal-regulated kinase; RET = a relevant receptor tyrosine kinase; PTEN = phosphatase and tensin homolog; Akt/CREB = signaling cascade in cell survival; HPA = hypothalamic–pituitary–adrenal axis; T3 = triiodothyronine; T4 = thyroxine; TSH = thyroid stimulating hormone; TC = thyroid cancer; ROS = reactive oxygen species; Nrf2 = Nuclear factor erythroid 2-related factor 2 (Prete et al. 2020; Hao et al. 2009; Maggisano et al. 2020; Unoki et al. 2016; Steinhaus et al. 2021; Marotta et al. 2020; Fiore et al. 2009; Boelaert 2009; Mohammadi-Bardbori and Rannug 2014; Buha et al. 2021; Tinkov et al. 2021; Ziros et al. 2013; Skalny et al. 2022; Wyatt et al. 2017; Pieper et al. 2014; Cebulska-Wasilewska et al. 2005)

The RAS/MAPK/PI3K pathway is activated by mercury exposure, which leads to the downstream activation of *MAPK* and *PI3K* (Hao et al. 2009; Maggisano et al. 2020; Unoki et al. 2016). These pathways are associated with cell growth, proliferation, and survival and are connected to activating mutations in TC (Prete et al. 2020). This suggests a role for MAPK/ERK pathway, as mercury exposure is linked to *MAPK* activation (Maggisano et al. 2020; Hao et al. 2009). *RET*/*PTC* fusion, *RET* activating mutations, and *BRAF* mutations are all observed in TC (Prete et al. 2020). This pathway results in ERK phosphorylation, essential for cell differentiation and growth.

Mercury exposure also inhibits *PTEN*, leading to *Akt*/*CREB* activation in the PTEN/Akt/CREB pathway (Unoki et al. 2016). This activation is associated with cell survival and growth, and *PTEN*-inhibiting mutations and *AKT1*-activating mutations are observed in TC (Prete et al. 2020). Another pathway impacted by mercury exposure is the endocrine/HPA axis, which causes decreased T3/T4 levels and elevated TSH levels (Marotta et al. 2020; Steinhaus et al. 2021). High TSH levels are associated with thyroid cell proliferation in TC (Boelaert 2009), highlighting the role of TSH-stimulated cell proliferation in disease progression (Table 2).

**Table 2:** Mercury emissions from different sources and corresponding global Thyroid cancer incidence and mortality rates

Region	Area	Stationary combustion sources	Industrial sources	Intentional use and product waste	SIR	SMR
North America	1.69 × 10^7^	32.4	34.8	41.1	12.4	0.3
Europe	5.53X10^6^	22.3	15.6	15.3	8.3	0.33
South Asia	5.07 × 10^6^	27.7	21.2	17.9	1.6	0.38
East Asia	1.16 × 10^7^	56.9	71.6	42	11.5	0.4
Southeast Asia	4.94 × 10^6^	7.9	14.1	16.4	4.4	0.63
Australia	8.06 × 10^6^	4.7	11.1	16.4	10.5	0.27
Africa	3.00 × 10^7^	33.7	50	80.7	2	0.62
Middle East	5.17 × 10^6^	3.7	8.3	10.2	8.6	0.83
Central America	5.21 × 10^6^	5.2	13.2	22.3	7.2	0.65
South America	1.53 × 10^7^	11.3	34.1	58.6	9.6	0.5
CIS countries	1.79 × 10^7^	27.3	42.9	40.4	7.3	0.4

Area: the area of the region in square kilometers. The quantity of pollutants (in tonnes/year) produced by three different environmental sources is shown. Additionally, age-adjusted standardized incidence rate (SIR) and standardized mortality rate (SMR) per 100,000 are displayed

Mercury exposure also increases reactive oxygen species (ROS) production (Skalny et al. 2022; Mohammadi-Bardbori and Rannug 2014; Buha et al. 2021), activating *Nrf2* signaling in the ROS/Nrf2 pathway (Tinkov et al. 2021; Buha et al. 2021). This pathway is implicated in PTC development and inhibits apoptosis, allowing cancer cells to evade programmed cell death (Ziros et al. 2013). Mercury-induced ROS production also leads to DNA damage and oxidative stress in the ROS/oxidative stress pathway, contributing to carcinogenesis and TC development (Wyatt et al. 2017). Lastly, in the DNA repair pathway, mercury exposure inhibits DNA repair proteins like PARP-1, DNA ligase III-alpha, and XRCC1, preventing DNA damage repair and promoting carcinogenesis (Pieper et al. 2014).

**Fig. 6:**
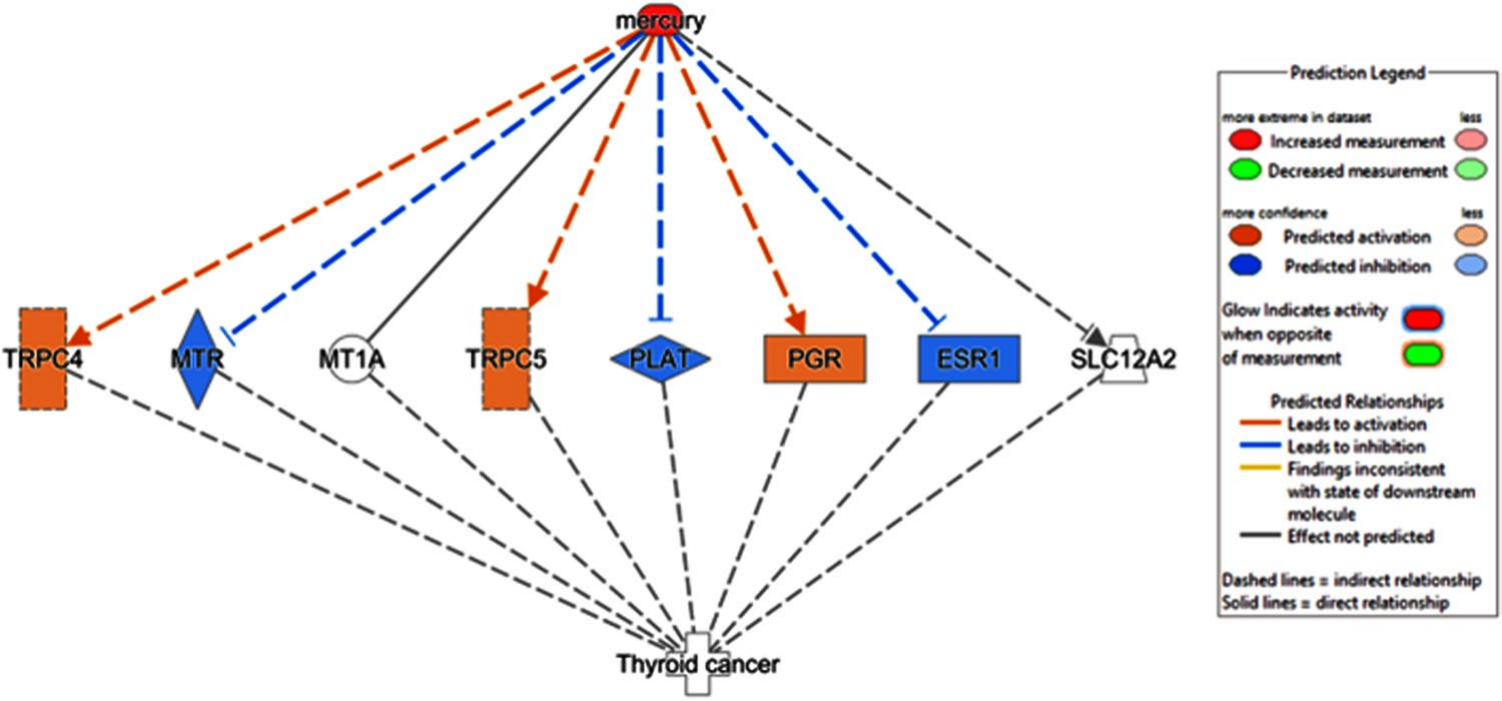
Molecular targets and pathways affected by mercury exposure in thyroid cancer development

Following our literature investigation, we performed a bioinformatics assessment using Ingenuity Pathway Analysis (IPA), which revealed that mercury affects multiple molecules involved in the development of TC. Mercury activates two plasma membrane ion channels, transient receptor potential cation channel subfamily C members 4 and 5 (*TRPC4* and *TRPC5*). Additionally, it influences the mRNA expression of two ligand nuclear receptors by decreasing the alpha estrogen receptor (*ESR1*) and increasing progesterone receptor A (*PGR*), as well as affecting the plasminogen activator peptidase (*PLAT*) in the extracellular space and inhibiting the cytoplasmic enzyme 5-methyltetrahydrofolate-homocysteine methyltransferase (*MTR*). Mercury chemically binds to metallothionein 1A (*MTA1*) in the cytoplasm and can activate and inhibit the transporter solute carrier family 12 member 2 (*SLC12A2*). These molecular targets are correlated with TC (Fig. 6).

The Catalogue Of Somatic Mutations In Cancer (COSMIC) database (https://cancer.sanger.ac.uk/cosmic) reveals mutations in human *TRPC4* (c.378 + 36501G > T), *TRPC5* (c.907G > A translating to p.A303T), *MTR* (c.128G > A translating to p.R43Q), *PLAT* (c.479T > A translating to p.L160Q), *PGR* (c.1955C > A translating to p.S652Y), *ESR1* (c.851-2646C > T), and *SLC12A2* (c.2451G > C translating to p.L817F) genes in TC cases (Tate et al. 2023). These molecules demonstrate a variety of relationships and responses to mercury exposure and TC. Understanding their associations with drugs and other molecular targets offers valuable insights into the effects of mercury on these molecules and their potential roles in the development of TC.

An overview of interconnected pathways provides insight into the complex interplay between mercury exposure and TC development. By understanding these mechanisms, we can better understand the role of mercury in thyroid carcinogenesis and develop effective strategies to address the risks and consequences of mercury-related TC. As knowledge of these pathways and their relevance to disease courses expands, it is crucial to translate this understanding into tangible clinical benefits for patients affected by TC in the context of mercury exposure.

## Mercury and thyroid cancer: the need for more research and future perspectives

This review aimed to identify connections between mercury exposure and pathway activation, exposure patterns, cellular impacts, and the carcinogenesis of TC. A literature review examined various routes of mercury exposure, including environmental exposures (such as proximity to volcanoes, polluted areas, and crematoriums) and occupational exposure (for dental professionals, miners, plant workers, and crematorium workers) (Taux et al. 2022; Fawell et al. 2005; Budnik and Casteleyn 2019; Pyle and Mather 2003; Bernhoft 2012; Tibau and Grube 2019). Dietary consumption through well water and fish is also potential risk factor for mercury exposure, although a significant relationship between dietary mercury and TC has not been established in the available literature as results have been inconclusive and limited to case–control designs (Fawell et al. 2005; Mahale et al. 2017; Wong et al. 2022; Nogara et al. 2019; Kim et al. 2020; Glattre et al. 1993; Kolonel et al. 1990; Hong and Myung 2023). The identification of these unique populations as susceptible to increased mercury exposure provides direction for future cohort studies which may help strengthen associations between mercury and TC or other health conditions.

The putative associations between mercury and thyroid carcinogenesis were also examined at the cellular and biochemical levels. Insults along the RAS, MAPK, and PI3K signaling cascades have been reported in myriad cell lines following mercury exposure (Hao et al. 2009; Maggisano et al. 2020; Unoki et al. 2016). Mutations in various genes identified in TC (Prete et al. 2020) have downstream effects comparable to and mirroring those that follow mercury-induced signaling and protein mercuriation. The formation of ROS following mercury exposure in cells has been observed, with impacts on DNA damage and repair, inhibition of apoptosis, and cellular proliferation (Skalny et al. 2022; Mohammadi-Bardbori and Rannug 2014; Buha et al. 2021; Tinkov et al. 2021; Ziros et al. 2013; Wyatt et al. 2017; Pieper et al. 2014; Cebulska-Wasilewska et al. 2005). The repercussions of cellular exposure to mercury have been established in numerous tissues, often resembling processes implicated in promoting thyroid carcinoma. Mercury's disruption of the thyroid-HPA axis is well established, with evidence of resultant elevations of TSH and pro-proliferative impacts on thyroid cells (Marotta et al. 2020; Steinhaus et al. 2021; Boelaert 2009). These findings provide a molecular basis for the role of mercury in thyroid carcinogenesis, suggesting a potential role for mercury exposure in eventual TC development. Some studies identified the carcinogenic impacts of mercury in thyroid cells, but many employed non-thyroid cell lines. Identifying these various points of convergence between mercury-induced carcinogenesis and the formation of sporadic TC will facilitate further study of these insults in thyroid tissue and the identification of causal cellular impacts.

**Fig. 7:**
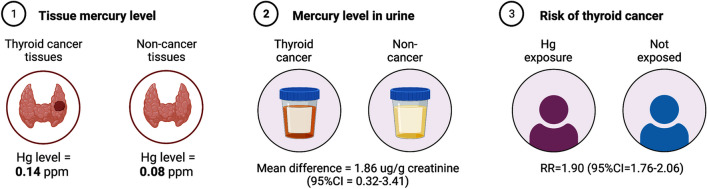
Summary of the results related to thyroid cancer and mercury levels (1) in thyroid tissues and (2) in urine samples compared to those with normal thyroid and (3) Risk of TC on mercury exposure

In our quantitative data analysis on tissue mercury levels and TC, our findings indicate a significant relationship between these variables. In patients with TC, elevated levels of mercury were discovered in both thyroid tissue and urine compared to those with normal thyroids, as summarized in Fig. 7 (Kim et al. 2022; Zaichick 2022; Correia et al. 2020; Zhang et al. 2019; Liu et al. 2021; Chung et al. 2016; Zaichick et al. 1995; Zidane et al. 2019). Individuals with elevated urine mercury exhibited a higher relative risk of developing TC (Kim et al. 2022; Liu et al. 2021; Zhang et al. 2019), further substantiating mercury as a crucial contributor to thyroid carcinogenesis. Although the power of our study is low, this provides a strong basis for the association between higher tissue mercury levels and TC. In our analysis of locoregional mercury emissions and TC incidence, we found some regions where high mercury emissions were correlated with high rates of TC and vice versa, however these results were inconsistent.

Our next steps and future directions must use more quantitative data and cohort studies to establish a stronger relationship between mercury and TC. Future studies are recommended to examine the incidence and prevalence of TC among populations close to sources of elevated environmental mercury, such as recently active volcanoes and high levels of mercury pollution. Population-based research for other key higher-risk exposure groups identified in this review may be of interest, including people with dental amalgam fillings containing mercury, people living near crematoriums, occupationally exposed groups, and people with high dietary mercury exposure. Moreover, considering the feasibility of incorporating "physiologically based toxicokinetic (PBTK)" modeling in future studies to elucidate the specific mechanisms behind mercury's impact on thyroid tissue is highly recommended. This approach could enhance our understanding of the relationship between mercury exposure and thyroid cancer development.

Ultimately, with further research establishing the potential associations between mercury and TC, there may be an increased public awareness of mercury exposures and risks, promoting improved health and ideally better TC outcomes. With emerging advancements in personalized medicine, knowledge of mercury exposure may eventually help in the guidance of targeted thyroid cancer preventative and early-intervention guidelines for identified at-risk individuals. Thus, further study is warranted to elucidate whether these associations exist for various routes of mercury exposure and to quantify what levels of mercury, if any, are safe for consumption (mainly through seafood), inhalation, and cosmetic products.

### Study limitations

The present work has some limitations. Our literature review uncovered many studies establishing an association between various routes of mercury exposure and tissue mercury levels, however there was sparse literature establishing an association between environmental mercury levels or occupational exposure to mercury and TC. Additionally, the health effects of mercury are likely dose dependent (Rice et al. 2014), which further complicates the ability to establish associations with TC development and the harms of mercury through different routes of exposure.

The effects of dietary mercury are challenging to study in humans. While seafood consumption can provide an approximation of dietary mercury intake, in the studies we reviewed, it represented only approximately 25% of overall caloric intake (Wong et al. 2022; Nogara et al. 2019; Kim et al. 2020). Measurements of total dietary mercury intake can be highly variable, necessitating quantitative control efforts. The absence of prospective or retrospective cohort studies investigating dietary mercury also restricts the impact of this review (Hong and Myung 2023). With the predominance of case–control studies, we cannot draw definitive conclusions.

The paucity of quantitative data investigating these relationships also constrains our findings. Among the studies found, there are discrepancies in the tissue reported, units of measurement (ug/L vs. ug/ g creatinine), and how data was reported. These studies do not always stratify between TC and goiter, allowing confounding. Additionally, some studies involved volcanic and occupational exposures (Skalny et al. 2022; Taux et al. 2022; Budnik and Casteleyn 2019; Pyle and Mather 2003; Bernhoft 2012; Sundseth et al. 2017; Edwards et al. 2021; Gworek et al. 2020; Tibau and Grube 2019). These may be problematic as they likely have numerous confounding variables due to concomitant exposure to chemicals and other heavy metals with known or suspected carcinogenic effects. These study designs also do not allow for assessing a dose–response relationship between mercury and thyroid cancer. Furthermore, the known diurnal variations in urine mercury excretion may contribute to the inconsistencies in our quantitative results (Calder et al. 1984).

When evaluating the associations between mercury emissions and locoregional TC incidence, there were significant limitations based on the available information. First, there were minor differences in the specific regional designations between the UNEP report and the GLOBOCAN database. The UNEP report also only accounts for anthropogenic mercury pollution and does not account for natural sources of mercury emissions such as volcanoes, cultural or dietary practices, and genetic factors. These confounders may be the reason for the inconsistencies in any potential associations between high mercury emissions and thyroid cancer incidence.

## Conclusions

The current study consolidates evidence to suggest that mercury exposure may be a potential risk factor for TC. The quantitative analysis shows a potential relationship between tissue mercury and TC based on existing research, but it was not statistically significant. Further research is needed, in particular, for some population sub-groups of interest which have documented or theorized mercury exposure including locoregionally exposed groups (e.g. those living in proximity to volcanoes, highly polluted areas, and crematoriums) and occupationally exposed groups (e.g. gold miners, crematorium workers, and dental professionals) to quantify this risk and elucidate mercury's role compared to other toxins and confounders, which act as modifying factors in carcinogenesis.

## Data Availability

The data presented in this study are available in the manuscript.
